# Cell line-dependent variability in HIV activation employing DNMT inhibitors

**DOI:** 10.1186/1743-422X-7-266

**Published:** 2010-10-13

**Authors:** Guerau Fernandez, Steven L Zeichner

**Affiliations:** 1 Center for Cancer and Immunology Research, Children's Research Institute, Children's National Medical Center, Washington DC, USA; 2 Departments of Pediatrics and Microbiology, Immunology, and Tropical Medicine, George Washington University School of Medicine, Washington DC, USA

## Abstract

Long-lived reservoirs of Human Immunodeficiency Virus (HIV) latently infected cells present the main barrier to a cure for HIV infection. Much interest has focused on identifying strategies to activate HIV, which would be used together with antiretrovirals to attack reservoirs. Several HIV activating agents, including Tumor Necrosis Factor alpha (TNFα) and other agents that activate via NF-kB are not fully effective in all latent infection models due to epigenetic restrictions, such as DNA methylation and the state of histone acetylation. DNA methyltransferases (DNMT) inhibitors like 5-aza-2'deoxycytidine (Aza-CdR) and histone deacetylase (HDAC) inhibitors like Trichostatin A (TSA) have been proposed as agents to enhance reactivation and have shown activity in model systems. However, it is not clear how the activities of DNMT and HDAC inhibitors range across different latently infected cell lines, potential models for the many different latently infected cells within an HIV patient. We determined HIV activation following treatment with TNFα, TSA and Aza-CdR across a range of well known latently infected cell lines. We assessed the activity of these compounds in four different Jurkat T cell-derived J-Lat cell lines (6.3, 8.4, 9.2 and 10.6), which have a latent HIV provirus in which GFP replaces Nef coding sequence, and ACH-2 and J1.1 (T cell-derived), and U1 (promonocyte-derived) cell lines with full-length provirus. We found that Aza-CdR plus TNFα activated HIV at least twice as well as TNFα alone for almost all J-Lat cells, as previously described, but not for J-Lat 10.6, in which TNFα plus Aza-CdR moderately decreased activation compared to TNFα alone. Surprisingly, a much greater reduction of TNFα-stimulated activation with Aza-CdR was detected for ACH-2, J1.1 and U1 cells. Reaching the highest reduction in U1 cells with a 75% reduction. Interestingly, Aza-CdR not only decreased TNFα induction of HIV expression in certain cell lines, but also decreased activation by TSA. Since DNMT inhibitors reduce the activity of provirus activators in some HIV latently infected cell lines the use of epigenetic modifying agents may need to be carefully optimized if they are to find clinical utility in therapies aimed at attacking latent HIV reservoirs.

## Findings

Despite the effectiveness of Highly Active Antiretroviral Therapy (HAART) for Human Immunodeficiency Virus type 1 (HIV-1) infection, patients cannot be cured due to the persistence of long-lived reservoirs of cells latently infected with HIV ([1-5] and reviewed in [6-11]). Much interest has focused on attacking this reservoir of HIV latently infected cells. A potentially useful strategy, sometimes termed "shock and kill" [12], aims to attack the latent reservoir treating patients with HIV-activating agents to stimulate HIV replication in the latently infected cells while blocking new infection of cells with antiretrovirals.

One activation approach employs agents like phorbol esters (e.g 12-O-Tetradecanoylphorbol-13-acetate (TPA), prostratin) [13], interleukins (IL-2, IL-7) [14-18] and cytokines (e.g. tumor necrosis factor alpha (TNFα)) [19] that directly activate HIV gene expression via well-known transcriptional activation pathways, like NF-κB, but many such agents are toxic or incompletely effective *in vivo *[14,16,20,21]. Other approaches target the provirus' epigenetic environment, employing histone deacetylase (HDAC) inhibitors (HDACIs) like trichostatin A (TSA) [22,23], suberoylanilide hydroxamic acid (SAHA) [22,24], sodium butyrate [25,26], and valproic acid [27,28], and/or DNA methyltransferase (DNMT) inhibitors (DNMTIs) like 5-aza-2'deoxycytidine (Aza-CdR) [29], with some strategies combining approaches [30] (and reviewed in [6,31]). In addition, to increase the efficiency of viral activators, combining the use of activators with compounds aimed at limiting the toxicity of the activator, as shown with buthionine sulfoximine [32] may play a major role in optimizing treatment strategies.

Nucleosomes occupy specific positions on the HIV LTR [22,33,34], with the nucleosome occupying the position termed 'nuc-1' at a regulatory region of the LTR 610 to 720 bp 3' to the transcription start site having significant inhibitory effects on HIV expression. Chromatin condensed around nuc-1 in its deacetylated form presents a block to HIV expression. Following hyperacetylation and chromatin remodeling, mediated by the recruitment of histone acetylases by transcription factors binding directly or indirectly to the LTR, or by Tat, the nuc-1 block is removed [22,33,35-37]. HDACIs, by shifting histones to a more acetylated state facilitate remodeling and removal of the block [38,39]. DNMTIs likely activate HIV because, at least in some cells, the LTR contains two CpG islands (particularly island 2) that can be hypermethylated and the hypermethylated DNA can recruit methyl-CpG binding domain (MDB) protein family members, notably MDB2. MDB2 recruited to the LTR can serve a bridging function between DNA and chromatin-modifying factors, such as HDACs [29]. Several studies linked retroviral promoter CpG methylation with transcription inactivation [40-42] and cellular gene silencing [43]. DNMTIs can moderately activate HIV alone, but in some systems they significantly enhance agents, like TNFα, that directly activate the HIV LTR, probably because activation is limited due to proviral DNA hypermethylation [29,44,45].

While DNMTIs and HDACIs significantly enhance HIV activation in some latently infected cells, their ability to enhance activation across a wider range of latently infected cells is currently unknown. The breadth of activating ability is important, since complete or close-to complete eradication of latently infected cells may be required to cure HIV infection. To better assess the breadth of activity of activators representative of both direct activators and activators acting via epigenetic effects we surveyed the ability of three prototypical HIV activating agents, the DNMTI Aza-CdR (2.5 μM), the HDACI TSA (1.5 μM), and TNFα (10 ng/ml), activating via NF-κB, in a panel of widely used HIV latently infected model cell lines, including the J-Lat series derived from Jurkat T-cells [46], J1.1 [47], also a Jurkat derivative, ACH-2 [48], derived from the A3.01 T lymphocytoid cell line, and U1 [49], derived from the U937 promonocytic cell line. The concentrations used for TSA and TNFα were optimized to obtain the best activation profile in combination with Aza-CdR. A dose response curve with J-Lat 6.3 cells was performed to determine the minimum concentration required where the activation reached a plateau (data not shown). We employed this plateau concentration in subsequent studies to assess the effects of combined treatments with different agents used at their maximally effective concentrations, since the object of any latency activation strategy would be to determine how to achieve a maximal degree of provirus activation.

J-Lat cells harbor an HIV provirus containing the Green Fluorescent Protein (GFP) ORF instead of *nef *and a frameshift mutation in *env *[46]. With GFP under the control of the HIV LTR, activation is conveniently monitored via flow cytometry. HIV production in J-Lat cells can also be determined by p24 antigen (p24) quantification. Different J-Lat lines exhibit different activation patterns when treated with TNFα, likely due to different integration sites and accompanying epigenetic states [46]. J-Lat cells have contributed significantly to latency studies, including the identification of the methyl-CpG binding domain protein 2 (MBD2), a transcriptional repressor that binds to methylated DNA, as a regulator of HIV latency, which suggested that DNMTIs like Aza-CdR might activate latent provirus [29]. Moreover, LTR demethylation, after Aza-CdR treatment, correlated with a synergistic increase of proviral activation when combined with TNFα in J-Lat 6.3, 8.4, 9.2 and 15.4. ACH-2, U1 and J1.1 harbor full-length proviruses, without GFP replacing coding sequence (although J1.1 has defects in signalling through CD3, ACH-2 has a point mutation in the Tat responsive element (TAR) [50], and U1 contains mutations in Tat [51]). Flow cytometric evaluation of activation is not possible in these lines, but they can be monitored using assays for HIV gene products (e.g. p24).

**Figure 1:**
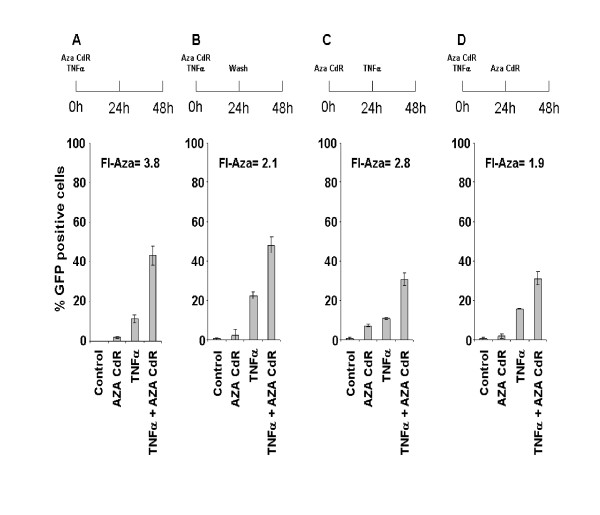
**Optimization of J-Lat 6.3 cells activation by TNFα/Aza-CdR**. HIV latently infected J-Lat 6.3 cells that contain the GFP reporter gene were (A) treated with Aza-CdR (2.5 μM) and TNFα (10 ng/ml) for 48 h, (B) were treated with Aza-CdR plus TNFα and then washed at 24 h, (C) treated with Aza-CdR followed by addition of TNFα after 24 h of Aza-CdR treatment, and (D) treated with Aza-CdR plus TNFα for 24 h, followed by the addition of fresh Aza-CdR. Cells were cultured in RPMI (Atlanta Biologicals, Lawrenceville, GA) with 10% FBS (Invitrogen, Carlsbad, CA). Two million cells were seeded with 3 ml medium in six well plates and treated with Aza-CdR (2.5 μM), TNFα (10 ng/ml) or both compounds combined. After 48 h cells were transferred to a 15 ml conical tube and spun down at 1400 rpm for 7 min. The supernatant was discarded and cell pellets were resuspended and were washed twice with chilled 1× PBS and fixed in 2% formaldehyde for FACS analysis using a FACScalibur cytometer (BD Biosciences, San Jose CA) and analyzed with Cell Quest Pro software (BD Biosciences, San Jose CA). GFP positive cells from the live population, defined by forward versus side scatter gating, were quantified. 20.000 events per treatment condition were analyzed. Results are the mean ± standard deviation (SD). The TNFαAza-CdR/TNFα (FI-Aza) ratio was calculated and used to evaluate the optimum time of activator addition.

Before embarking on a survey of the activators across cell lines, we undertook further optimization experiments, initially performing order-of-addition experiments using J-Lat 6.3 (Fig.1), in which Aza-CdR had been shown to enhance activation mediated by TNFα [29], since inhibiting DNA methylation well before addition of the direct transcriptional activator TNFα could be required for maximal activation. Detection and quantification of GFP positive cells from a live-gated population was performed at 48 h using a FACScalibur cytometer (BD Biosciences, San Jose CA), analyzed with Cell Quest Pro software (BD Biosciences, San Jose CA). As has been previously reported, we found that Aza-CdR alone did not activate [29], but TNFα alone produced significant increases in GFP positive cells, and Aza-CdR enhanced activation with TNFα treatment. We calculated the fold increase of activation due to Aza-CdR, that is the activation observed with TNFα plus Aza-CdR divided by the activation with TNFα alone (TNFα+Aza-CdR/TNFα, (FI-Aza)), which expresses the fold increase due to the addition of Aza-CdR. FI-Aza showed that the greatest activation occurred when J-Lat 6.3 cells were treated with both compounds simultaneously (FI-Aza:3.8). Although there were differences in the extent of activation that depended on the order of addition, Aza-CdR always enhanced activation of J-Lat 6.3 by TNFα, with FI-Aza ranging between 3.8 and 1.9.

**Figure 2:**
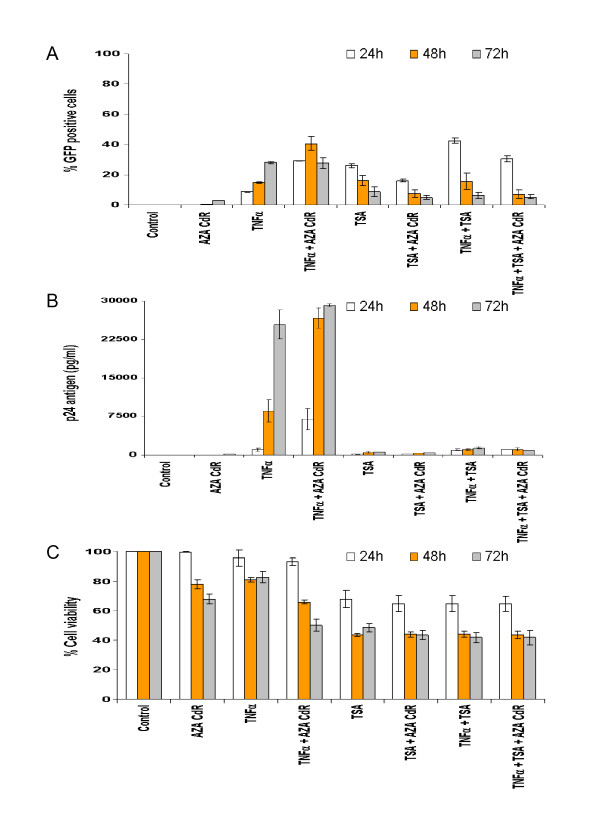
**Time-course activation by TNFα and TSA alone or in combination with Aza-CdR in J-Lat 6.3 cells**. A. GFP positive cell quantification by FACS analysis at 24, 48 and 72 h post-induction for all treatments and treatment combinations using J-Lat 6.3 cell line. GFP positive cells from the live population, defined by forward versus side scatter gating, were quantified. 20.000 events per treatment condition were analyzed. B. p24 antigen production determined using an enzyme-linked immunosorbent assay (ELISA) (Perkin Elmer, Waltham, MA) for all post-induction time points and treatments as for panel A. C. Cell viability of the different treatments and treatment combinations for all time points determined with MTS assay (Promega, Madison, WI). Final activator compound concentrations were, for Aza-CdR (2.5 μM), TNFα (10 ng/ml) and TSA (1.5 μM). Results are the mean ± standard deviation (SD).

**Figure 3:**
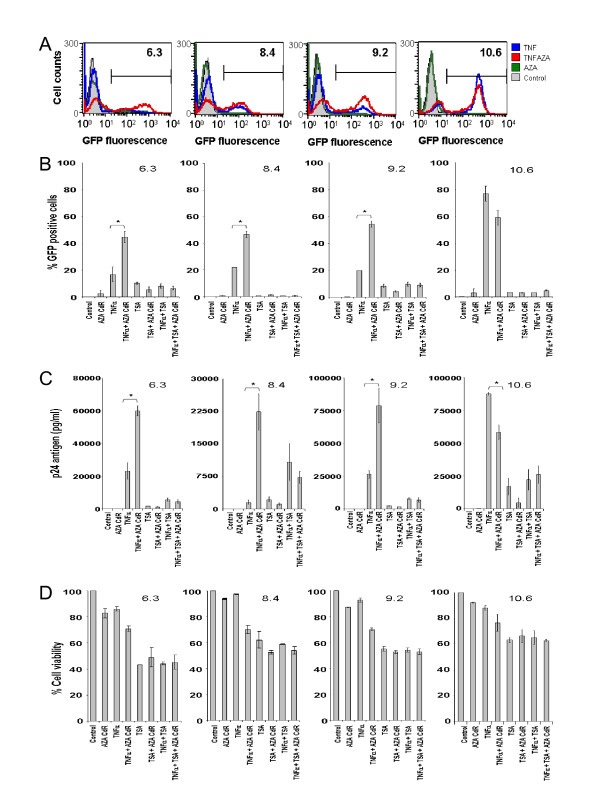
**HIV activation by TNFα and TSA alone or in combination with Aza-CdR in four J-Lat cell lines**. A. GFP positive cell quantification by FACS analysis at 48 h post-induction of J-Lat 6.3, 8.4, 9.2 and 10.6 cell lines treated with TNFα or Aza-CdR alone or in combination. 20.000 events from the live population defined by forward versus side scatter gating were analyzed. B. Proviral activation as determined by percent GFP positive cells for the different treatments and treatment combinations in the four J-Lat cells (J-Lat 6.3, 8.4, 9.2 and 10.6 indicated on each panel). C. p24 antigen production for all treatments and treatment combinations at 48 h post-induction. D. Cell viability of the different treatments and treatment combinations determined with an MTS assay (Promega, Madison, WI) at 48 h. Cells were cultured in RPMI with 10% FBS. Two million cells were seeded and treated with the different compounds for 48 h. The compound order of addition was determined as described in Fig. 1: agents were added at time 0 h, with no further additional steps. After 48 h, cells were washed twice with chilled 1× PBS and fixed in 2% formaldehyde as in the FACS analysis described in the Fig. 1 legend. At the same time, supernatants from the different treatment conditions were stored at -80°C until further use. p24 antigen was determined using ELISA (Perkin Elmer, Waltham, MA) from the stored supernatants. Final activator compound concentrations were, for Aza-CdR (2.5 μM), TNFα (10 ng/ml) and TSA (1.5 μM). Results are the mean ± standard deviation (SD). Statistical analysis (Student's t-test) was performed using the STATA software package (StataCorp LP, College Station, TX), * p ≤ 0.05.

To determine when Aza-CdR most enhanced TNFα or TSA activation, a time-course experiment was performed using J-Lat 6.3 cells. Percent GFP positive cells using FACS analysis (Fig. 2A) and p24 production using an enzyme-linked immunosorbent assay (ELISA) (Perkin Elmer, Waltham, MA) (Fig. 2B) were determined for all time points and treatment conditions. Aza-CdR in combination with TNFα reached its maximum effect at 48 h post-induction when per cent GFP positive cells where quantified. Moreover, p24 production did not increase significantly beyond 48 h, losing Aza-CdR's enhancing effect when combined with TNFα at later time points. Due to the lack of synergy between TSA and Aza-CdR we determined 48 h to be the best time point to analyze further data based on TNFα plus Aza-CdR results. Cell viability for all time points and treatment conditions (Fig. 2C) were performed using MTS assay (Promega, Madison, WI). TNFα alone does not strongly activate J-Lat 6.3, 8.4 and 9.2 cells, as shown in Fig. 3A, and as reported [29]. TNFα can, at most, achieve 20% activation in these three cell lines. In contrast, in J-Lat 10.6, TNFα activates ~80% of the cells, which enabled us to determine the effect of combining Aza-CdR with TNFα and TSA when the provirus is highly activated by TNFα. Surprisingly, the combination of Aza-CdR and TNFα did not increase or maintain activation in J-Lat 10.6, but instead moderately decreased activation (p ≤ 0.1) (Fig. 3B) in per cent GFP positive cells while activation from the combination of Aza-CdR and TNFα was significantly lower when p24 was determined (p ≤ 0.05) (Fig. 3C). The reduction of activation in J-Lat 10.6 suggested that Aza-CdR could have, in some latently infected cells, a detrimental effect when combined with an activator, particularly when activation via other pathways, such as those stimulated by TNFα, is great (Fig. 3C). Cell viability did not differ between TNFα and Aza-CdR, alone or in combination (Fig. 3D), so cell toxicity could not account for the observations. Of note, we did not detect a synergistic effect when TNFα and TSA were combined when percent GFP positive cells were quantified. As shown in the time-course experiment in Fig. 2 this synergy can be detected in earlier time points but not at 48 h post-induction when Aza-CdR reaches maximal effect. When p24 production is determined, we only could see this TSA-TNFα synergy in J-Lat 8.4 cells because TNFα activation in this cell line is strongly blocked (Fig. 3C).

**Figure 4:**
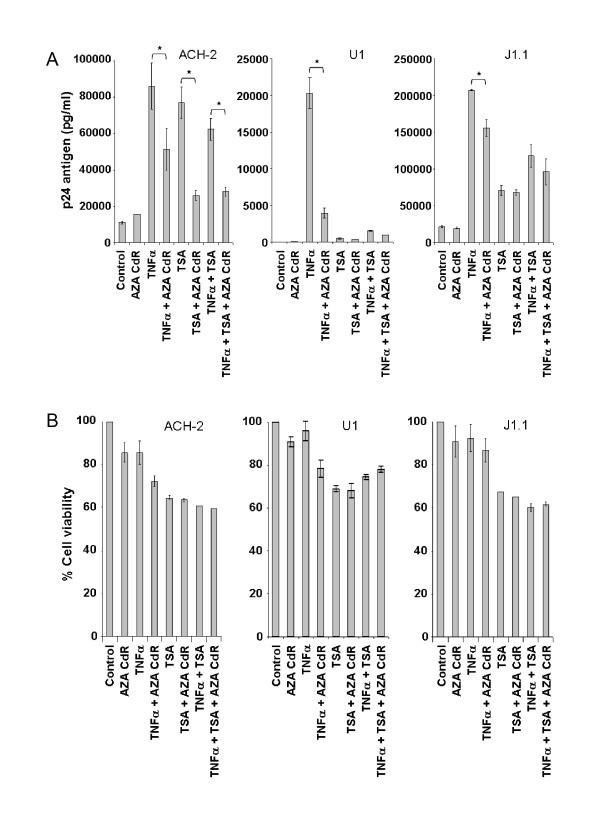
**HIV activation by TNFα and TSA in ACH-2, U1, and J1.1 latently infected cell lines with or without Aza-CdR**. A. p24 antigen production in ACH-2, U1 and J1.1 cell lines for all treatments and treatment combinations at 48 h post-induction. Cells were cultured in RPMI with 10% FBS. 2 million cells were treated with the different compounds for 48 h. The compound order of addition was determined in Fig. 1. After 48 h, cells were pelleted at 1400 rpm for 7 min and discarded. Supernatants from the different treatment conditions were stored at -80°C until further use. p24 antigen was determined using ELISA (Perkin Elmer, Waltham, MA) from the stored supernatants. B. Cell viability of the different treatments and treatment combinations shown in panel A, determined with an MTS assay (Promega, Madison, WI) at 48 h post-treatment. Compound final concentrations of the activators were Aza-CdR (2.5 μM), TNFα (10 ng/ml) and TSA (1.5 μM). Results are the mean ± standard deviation (SD). Statistical analysis (Student's t-test) was performed using STATA software package (StataCorp LP, College Station, TX), * p ≤ 0.05.

To assess how the combinations of Aza-CdR, TNFα, and TSA affect HIV activation in other HIV latently infected cells, we studied the ability of these agents, alone and together, to activate HIV in ACH-2, U1 and J1.1 cells (Fig. 4A), other lines in which TNFα strongly activates HIV expression [52]. Since these cells harbor a latent HIV provirus that does not have *nef *replaced with GFP, we assessed activation by measuring p24 48 h after treatment with the activators using ELISA (Perkin Elmer, Waltham, MA). TNFα powerfully activated HIV expression in these cells, as previously described, but as we observed with J-Lat 10.6, treating ACH-2, U1 and J1.1 cells with Aza-CdR significantly decreased activation by TNFα, from 23% (J1.1, p ≤ 0.05) to 40% (ACH-2, p ≤ 0.05), to 75% (U1, p ≤ 0.05). In ACH-2 cells, but not in any other of the cell lines studied, Aza-CdR also significantly decreased activation following treatment with TSA alone (57%, p ≤ 0.05), or TSA+TNFα (45%, p ≤ 0.05). Impaired cell viability, as with J-Lat cells, could not explain this reduction in activation (Fig. 4B). Thus, in certain circumstances, a DNMTI can decrease activation by agents acting both through the NF-κB pathway and mediated by an HDACI. However, a decrease in activation produced by Aza-CdR was not observed in all the non-J-Lat cell lines under all conditions. In J1.1 cells, Aza-CdR did not decrease activation by TSA or TSA plus TNFα. Overall, the ability of the DNMTI Aza-CdR to help activate HIV replication (or to inhibit the activation produced by other agents) exhibited a strong cell line dependence: In U1 and J1.1 cell lines, Aza-CdR inhibited activation by TNFα, but not by TSA or TNFα plus TSA, while in ACH-2 cells Aza-CdR inhibited activation by both TNFα and TSA when used alone or in combination. Table 1 summarizes the effect of Aza-CdR and TNFα when added simultaneously in all latently infected cells tested, highlighting that the effects due to the combination of agents may be detrimental or helpful, depending on the cell line studied. The data presented in this report complements the findings by Kauder et al. [29] and Blaskova et al. [44] regarding the effects of Aza-CdR in activating latently infected cells. In those studies, cell lines H12 and 2D12 [44] and all the J-Lat clones except for A2 clone [29] showed increased activation with Aza-CdR used in combination with TNFα in comparison with TNFα alone (the A2 clone showed the same levels of activation with or without Aza-CdR). The detrimental effect we observed with Aza-CdR in certain latently infected cell lines induced by viral activators like TNFα (ACH-2, J1.1 and U1) highlights the complexity of HIV reactivation, the importance of studies utilizing a broad range of cell lines, and the broad range of effects that may be observed in different infected cells.

**Table 1:** Effect of AZA-CdR in TNFα HIV latently infected cell activation

		**Control**	**AZA-CdR**	**TNFα**	**TNFα + AZA-CdR**
		
J-Lat	6.3	0.0 ± 0.0	62 ± 0.6	23136 ± 5060	60090 ± 3090
	8.4	0.0 ± 0.0	0.0 ± 0.0	1613 ± 485	22427 ± 4215
	9.2	30 ± 0.8	83 ± 4	26490 ± 2798	78921 ± 13057
	10.6	80 ± 10	190 ± 48	87578 ± 1071	58164 ± 5693
					
	ACH-2	11083 ± 670	15445 ± 12	85997 ± 12654	51347 ± 11353
	U1	12 ± 1	44 ± 5	20305 ± 2150	3943 ± 687
	J1.1	21475 ± 1475	19577 ± 1335	207468 ± 890	155646 ± 11578

Mean p24 (pg/ml) ± standard deviation

The differential effects on HIV activation of epigenetic agents may not be entirely unexpected, since the chromatin structure of the LTR can differ for the proviruses integrated into one cell line or another [53]. It will require much additional work to understand the mechanisms responsible for the differential responses to epigenetic activators, but a few potential explanations may be considered. Aza-CdR may have complicated effects, since the activation of some genes may inhibit others [54-56], so differences in the methylation and hence activation state of cellular genes in the latently infected cell lines could account for the differences in activation observed with Aza-CdR: In J-Lat 6.3, 8.4 and 9.2 cells, Aza-CdR may have direct effects, leading to the demethylation of the LTR, enhancing activation, while in the other lines Aza-CdR may help activate other cellular genes that directly or indirectly inhibit HIV activation, since differential expression of certain cellular genes can be associated with the maintenance of latency [57,58].

In HIV infected patients, the latently infected cells harbor many different proviruses. While the frequency of latently infected cells in the periphery is low, perhaps 1 in 10^6 ^cells, the total number of latently infected cells within a patient has been estimated to be as high as 10^6^-10^7 ^total cells [59]. The latently infected lines we examined may not be completely representative of latently infected cells as they exist *in vivo*, but the great potential variability in host cell chromosomal location and epigenetic and transcriptional environments of proviruses from as many as 10^6^-10^7 ^total latently infected cells suggests that, at least for some latently infected cells that exist *in vivo*, DNMTIs and HDACIs may inhibit HIV activation, as we describe here. While it is clear that DNMTIs and HDACIs offer promise as agents to attack the reservoir, our findings suggest that it may be necessary to carefully optimize HIV activation strategies so that some treatment or combination of treatments is active across large numbers of latently infected cells. For effective clinical applications, fairly elaborate combinations of activators and co-activators may be required to assure that essentially all proviruses are induced into active replication.

## Abbreviations

Aza-CdR: 5-aza-2'deoxycytidine; DNMT: DNA methyltransferase DNMTI: DNMT inhibitor; GFP: green fluorescent protein; HAART: highly active antiretroviral therapy; HDAC: histone deacetylase; HDACI: HDAC inhibitor; HIV-1: Human Immunodeficiency Virus type I; LTR: Long Terminal Repeat; MTS: 3-(4,5-dimethylthiazol-2-yl)-5-(3-carboxymethoxyphenyl)-2-(4-sulfophenyl)-2H-tetrazolium, inner salt; SAHA: suberoylanilide hydroxamic acid; TAR: Trans-activation response element; TNFα: tumor necrosis factor alpha; TPA: 12-O-tetradecanoylphorbol-13-acetate; TSA: trichostatin A

## Competing interests

The authors declare that they have no competing interests.

## Authors' contributions

GF carried out the experiments, data analysis and drafted the manuscript. SZ participated in the design of the study and data analysis, and drafted the manuscript. All authors read and approved the final manuscript.
